# Barriers to the Implementation of Local Anesthesia for Older Adults Undergoing Inguinal Hernia Repair

**DOI:** 10.1111/jgs.70402

**Published:** 2026-03-26

**Authors:** Don Mai, Benjamin A. Y. Cher, Nicole Lunardi, Melissa Thornton, Jocelyn G. Baker, Cameron Macdonald, Celette Sugg Skinner, Cynthia J. Brown, Elisa L. Marten, Konstantinos Makris, Maria Luisa Machado Heredia, Miles Berger, C. Munro Cullum, Simon Lee, Thai H. Pham, Courtney J. Balentine

**Affiliations:** 1 Department of Surgery, University of Wisconsin-Madison SMPH, Madison, Wisconsin, USA; 2 William S. Middleton Memorial VA Hospital, Madison, Wisconsin, USA; 3 Wisconsin Surgical Outcomes Research Group (WiSOR), Madison, Wisconsin, USA; 4 Department of Surgery, University of Texas Southwestern Medical Center, Dallas, Texas, USA; 5 VA North Texas Health Care System, Dallas, Texas, USA; 6 Qualitative Health Research Consultants, Madison, Wisconsin, USA; 7 Peter O’donnell Jr. School of Public Health, University of Texas Southwestern Medical Center, Dallas, Texas, USA; 8 Department of Internal Medicine, Louisiana State University School of Medicine, New Orleans, Louisiana, USA; 9 Department of Surgery, Baylor College of Medicine, Houston, Texas, USA; 10 Michael E. DeBakey Veterans Medical Center, Houston, Texas, USA; 11 Department of Anesthesiology, Duke University Medical Center, Durham, North Carolina, USA; 12 Department of Psychiatry, University of Texas Southwestern Medical Center, Dallas, Texas, USA; 13 Department of Population Health, University of Kansas Medical Center, Kansas City, Kansas, USA

**Keywords:** cognitive recovery, hernia repair, implementation, local anesthesia, older adults

## Abstract

**Background:**

Use of local anesthesia for inguinal hernia repair in older adults is recommended by society guidelines because of reduced postoperative cognitive dysfunction and improved functional recovery after surgery compared to general anesthesia. However, in practice, few inguinal hernia repairs in older adults are actually done under local anesthesia.

**Participants and Setting:**

Key stakeholders involved in inguinal hernia repair at Veterans’ Affairs hospital systems including patients, surgeons, anesthesiologists, and hospital leaders.

**Methods:**

We conducted semi-structured interviews and focus groups to identify barriers and facilitators to use of local anesthesia for inguinal hernia repair in older adults. Qualitative data were analyzed using Direct Content Analysis guided by the Capability, Opportunity, and Motivation (COM-B) framework.

**Results:**

We interviewed 40 Veterans aged ≥ 65 years who had undergone inguinal hernia repair at 3 hospitals, and we convened focus groups and interviews with 10 surgeons, 9 anesthesiologists, and 7 hospital leaders. We found patients were not consistently offered the opportunity to have shared decision-making conversations about the advantages and disadvantages of local versus general anesthesia because standard pre-operative care workflows did not allow for conversations incorporating perspectives of all stakeholders, especially anesthesiologists. Also, it was difficult to disentangle choice of anesthesia modality from choice of surgical approach. There were entrenched opinions among many surgeons about perceived advantages of minimally invasive surgery, which requires general anesthesia, without regard for the cognitive and recovery benefits of open surgery using local anesthesia. Finally, providers and hospital leaders highlighted how improved protocols for local anesthesia administration, increased buy-in from hospital leadership, and more high-quality evidence in support of local anesthesia were all necessary to increase its use.

**Conclusions:**

The findings serve as a roadmap for a multi-component plan to increase use of local anesthesia for inguinal hernia repair in older adults, with clear advantages for post-operative cognitive and functional recovery.

## 1 | Introduction

The U.S. population is rapidly aging, and soon there will be more people ≥ 65 years old than < 18 years of age [1]. Since patients ≥ 65 years old are more likely to experience postoperative morbidity, mortality, and disability than younger patients, and over 4 million U.S. patients ≥ 65 years old undergo surgery every year, it is critical to identify ways to improve postoperative outcomes for older patients [2–4]. One method that can potentially improve postoperative outcomes for older patients is to use local anesthesia as an alternative to general anesthesia [5, 6]. Use of local rather than general anesthesia is recommended by the joint American College of Surgeons and American Geriatrics Surgery guidelines for inguinal hernia repair in older adults because it may reduce postoperative cognitive dysfunction and improve functional recovery after surgery [7]. In particular, randomized trials have shown that using local anesthesia as an alternative to general anesthesia leads to outcomes that are at least as good or better than using general anesthesia and can significantly reduce costs of care [8–11]. Despite ample evidence supporting the use of local anesthesia for inguinal hernia repair, only 10%–15% of the 400,000 inguinal hernia operations in the US each year are done using local anesthesia [12, 13].

Since greater use of local anesthesia for hernia surgery in older adults could potentially improve outcomes and lower costs, we aimed to identify barriers and facilitators to increasing the use of local anesthesia from the perspectives of key stakeholders, including patients, surgeons, anesthesiologists, and hospital leadership. We conducted a qualitative study using interviews with Veterans and focus groups with physicians and hospital leaders, guided by the Capability, Opportunity, and Motivation (COM-B) model [14], which synthesizes multiple theories of behavior change.

## 2 | Methods

### 2.1 | Setting

We interviewed 40 Veterans aged ≥ 65 years old who had undergone inguinal hernia repair at a convenience sample of three participating VA sites with large surgical volume for inguinal hernia repair that perform the operation using both local and general anesthesia.

### 2.2 | Study Design

We conducted a qualitative survey using interviews with Veteran and focus groups with other stakeholders involved in inguinal hernia repair including surgeons, anesthesiologists, and hospital leaders. We asked all stakeholders to discuss facilitators and barriers to increasing the use of local anesthesia in hernia repair for older patients. The analysis was guided by the COM-B model, which synthesizes multiple theories of behavior change [14]. The results were reported in accordance with the EQUATOR network’s Standards for Reporting Qualitative Research [15]. The Institutional Review boards at the Dallas and Houston Veterans Affairs (VA) Hospitals and the University of Wisconsin-Madison approved this study.

### 2.3 | Screening & Recruitment

We approached Veterans at the time of initial surgical consultation in the general surgery clinic. Veterans were eligible for inclusion if they were ≥ 65 years old and had elective open or laparoscopic repair of unilateral, non-recurrent inguinal hernias. Veterans were excluded if they had cognitive deficits that prevented interview participation, if they were not fluent in English, or if surgery was for a recurrent hernia. We utilized a purposive sampling frame designed to recruit half of the sample from each site, to ensure that half of the Veterans recruited had surgery under local anesthesia and half under general, and that half were aged 65–74, and half were aged 75 or greater. Those who consented were referred to an independent qualitative research firm, which scheduled and conducted phone interviews. Veterans were compensated $50 upon interview completion. The firm also participated in data analysis and preparation of the manuscript.

Surgeons, anesthesiologists, and hospital leaders were recruited via email invitation from the PI and referred to the same firm to schedule virtual focus groups or 1:1 interviews. Surgeons and anesthesiologists were eligible for recruitment if their practices included inguinal hernia surgery. Hospital leaders were eligible if their hospitals or departments offered inguinal surgery. Per VA guidelines, compensation to employees was not permitted.

### 2.4 | Semi-Structured Interviews and Focus Groups

We conducted pilot interviews with four Veterans to test interview question comprehension and to revise the guide with the final version shown in File S1.

Interviews and focus groups with surgeons, anesthesiologists, and hospital leaders asked about their views on different approaches to anesthesia and explored individual and systems-level barriers and facilitators to use of local anesthesia. Focus groups and interviews with hospital leaders and providers took place over Zoom and lasted 1 hour. Veteran interviews ranged from 60 to 90 min in length and were conducted by phone. Audio was transcribed verbatim by an independent transcription firm and de-identified following HIPAA guidelines to ensure privacy and confidentiality.

### 2.5 | Use of the Capability, Opportunity, and Motivation (COM-B) Framework

COM-B is a commonly applied implementation framework that categorizes barriers and facilitators to individual and systems-level change into three primary categories: (1) Capability refers to individuals’ physical ability to perform tasks (e.g., ability to successfully provide pain control via injection of local anesthesia) and their psychological ability or belief in their capacity to accomplish tasks (e.g., uncertainty about using local because they’ve only done it once). (2) Opportunity describes the physical opportunities (e.g., facility workflows may make use of local anesthesia infeasible) or social opportunities (e.g., norms, values and beliefs) that facilitate or hinder practices. (3) Motivation refers to the factors that generate a desire to adopt or change practice (e.g., patients request use of local anesthesia or surgeons are motivated to reduce operative time and believe use of local anesthesia will accomplish this). Importantly, the framework captures both organizational and individual factors that drive practice and could be targeted in interventions designed to change practice.

### 2.6 | Data Analysis

Research assistants, the principal qualitative analyst, and the principal investigator utilized Directed Qualitative Content Analysis based on the COM-B framework to develop a codebook, which is presented in Files S2 and S3 [16, 17]. Directed Qualitative Content Analysis involves a combination of deductive and inductive data coding, in which an existing theoretical framework, in this case the COM-B, is used to create an initial set of coding categories, after which the research team develops a set of inductively-derived codes based on concepts emerging out of the data. Research Assistants applied all codes (see File S2) across all transcripts using NVivo qualitative analysis software [18]. Three research assistants coded all transcripts, commonly coding every fourth transcript to check for intercoder reliability and achieving a kappa score of 0.81, demonstrating strong agreement. The research team convened regular coding meetings to adjudicate differences in coding and to discuss emerging codes with the principal investigator.

Once all data were coded, the team reviewed NVivo queries of all utterances coded at each Com-B component, and identified overarching patterns through which Capability, Motivation and Opportunity acted as a barrier or facilitator to the use of local anesthesia in selected surgeries. These broad themes formed the basis of our results and are listed in Table 1.

## 3 | Results

**FIGURE 1 |:**
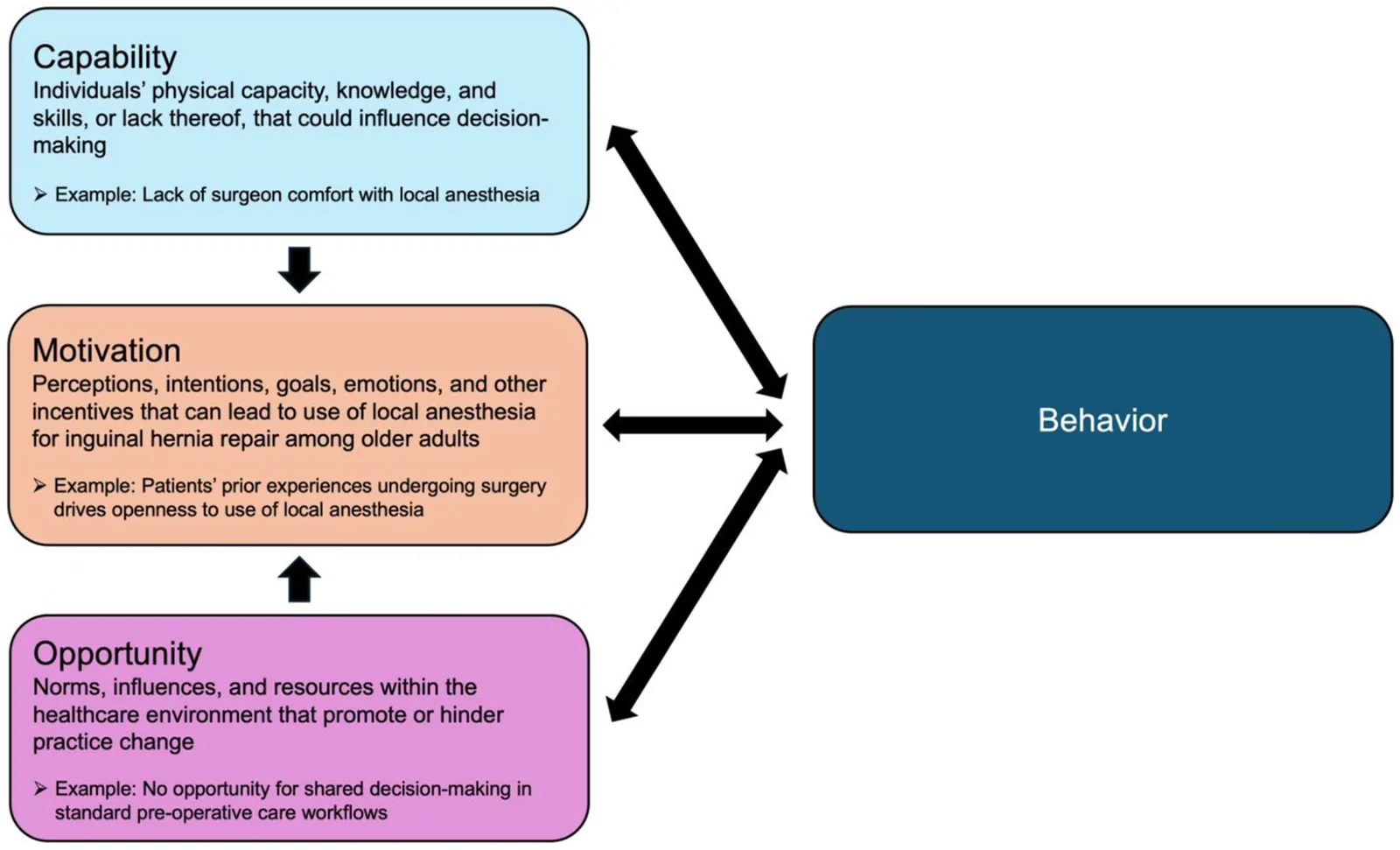
Example study themes within the Capability, Opportunity, and Motivation (COM-B) framework for behavior change.

We completed interviews with 40 Veterans, 10 surgeons, 9 anesthesiologists, and 7 hospital leaders. Themes emerging out of the COM-B codes and example quotes from patient and provider interviews are provided in Figure 1 and Table 1.

### 3.1 | Participant Characteristics

#### 3.1.1 | Patients

Among Veteran participants, mean age was 73 ± 5 years, 65% were White, 32.5% were Black, and all were men (Table 2). Most of the Veterans (95%) underwent open hernia repair, and 5% had robotic surgery. Local anesthesia was used for 47.5% of patients, and general anesthesia was used for 45% of patients. For 7.5% of patients, the anesthesia type was converted from local to general anesthesia during surgery.

#### 3.1.2 | Providers

Among hospital leaders, surgeons, and anesthesiologists, 88% were < 60 years old, 65% were white (65%), and 35% were Asian (35%) (Table 3). Most were men (69%), and 81% had completed fellowship training after residency. Overall, 86% of hospital leaders and 56% of anesthesiologists had been in practice for > 20 years, while 80% of surgeons had been in practice for < 20 years.

### 3.2 | Barriers and Facilitators to Use of Local Anesthesia for Inguinal Hernia Surgery

Below, we discuss the various barriers and facilitators identified by patients and surgeons. These are organized into domains defined by the COM-B framework.

#### 3.2.1 | Capability

##### 3.2.1.1 | Patients.

Many Veterans did not understand the advantages and disadvantages of local versus general anesthesia and/or did not understand the importance of anesthesia type. For example, some Veterans were unaware of the potential for greater cognitive decline after general anesthesia than after local anesthesia. For example, when one Veteran was asked about how he would feel if he had any changes in his thinking ability following hernia surgery, he responded as follows:

Let me plead ignorance here … Why, I mean, I don’t [mind] answering the questions, but why would you be asking questions about things that have absolutely nothing to do with hernia surgery?

Some Veterans had insight into their lack of understanding and expressed apprehension about this, recognizing there was nothing they could do besides blind trust in the surgeons and anesthesiologists to make the choice about anesthesia type. In some cases, Veterans were nervous about asking what could be perceived as “stupid questions,” feeling there was not much they could do beyond trusting their doctors to do the right thing.

On the other hand, some Veterans who understood the risks and benefits of both types of anesthesia were able to make informed decisions about their care. Some Veterans expressed a preference for general anesthesia because they feared experiencing pain during surgery or simply preferred to be unconscious. Others preferred local anesthesia because they were concerned that their advanced age and comorbidity burden put them at higher risk for complications with general anesthesia, and they felt this risk could be mitigated under local anesthesia.

##### 3.2.1.2 | Providers.

Surgeons who rarely used local anesthesia discussed how their lack of training made them less comfortable with the technique, while surgeons who frequently used local anesthesia expressed their comfort with doing so. Many surgeons mentioned they were less likely to use local anesthesia because they were worried about patients feeling pain during the procedure or being unable to remain still during the course of the surgery. Hospital leaders and anesthesiologists consistently stated that the principal factor limiting greater use of local anesthesia was the surgeon’s familiarity and comfort with this approach.

#### 3.2.2 | Opportunity

##### 3.2.2.1 | Patients.

Many patients reported that they were not offered an opportunity to learn about or discuss the merits of local versus general anesthesia during pre-operative consultations. Some Veterans recalled being told definitively that they were going to have their procedure under local anesthesia, even though they were nervous about this and had a specific preference for general anesthesia. One Veteran recalled being told forcefully that his comorbidities precluded use of general anesthesia, despite his concerns about having the operation under local anesthesia.

##### 3.2.2.2 | Providers.

Surgeons, anesthesiologists, and hospital leaders all highlighted the lack of standardized protocols for use of local anesthesia. They specifically noted it was unclear whether surgeons or anesthesiologists would perform blocks, and there were no protocols for timing and dosing of anesthetic medication. This confusion led to increased use of general anesthesia, which was easier to implement because of greater standardization and clear realms of responsibility between anesthesia and surgical providers.

Additionally, anesthesiologists reported limited opportunity to influence the decision between local and general anesthesia because they typically meet Veterans after surgery has been scheduled, and after the surgeon has already discussed the surgical and anesthesia plans. Consequently, even anesthesiologists who strongly believed in the benefits of local anesthesia for older adults felt they could not effectively advocate for its use when the surgeon had already told the patient they would be “completely asleep” during the procedure. For example, as one anesthesiologist stated:

I think it’s very important because the surgeon is the one who first sees the patient. And if they set up the expectation and the attitude about a surgery … we can discuss with the patient. But if the surgeon tells the patient, ‘we just have to put you to sleep,’ that’s what the patient expects. And when we present another idea to the patient, they’re not going to follow us.

Hospital leaders identified strong buy-in and support from institutional leadership as a necessary facilitator to increased use of local anesthesia, mentioning that the directive to use local anesthesia more frequently would need to come from those in positions of power.

#### 3.2.3 | Motivation

##### 3.2.3.1 | Patients.

Veterans who had undergone previous operations, especially if those were performed under local anesthesia, were more willing to consider using local for the hernia operation and were generally more flexible about the choice of anesthesia modality. Some older Veterans reported negative experiences with general anesthesia, such as urinary retention or nausea, which motivated them to seek out an alternative. One veteran was particularly concerned about the risk of cognitive decline:

If I went through the surgery and was not able to work, because I couldn’t function around machinery, I couldn’t think quick enough to respond, I could wind up hurting myself or injuring somebody else out there. You know, thousands and thousands of pounds of steel fall easily, and you don’t pay attention, and you can’t function, you can’t do your job. I think that’s a failure. That … is not acceptable.

This issue was also identified in the interviews with providers, who mentioned that patient preferences, which were often grounded in prior experiences undergoing surgery, could serve as a key barrier or facilitator to the use of local anesthesia depending on the nature of that prior experience. Anesthesiologists also mentioned how providing patients with a sense of psychological safety may help them feel more comfortable in their choice to undergo local anesthesia.

##### 3.2.3.2 | Providers.

Several surgeons expressed a strong preference for using a minimally invasive approach (either laparoscopic or robotic) for inguinal hernia repair, which necessitates use of general anesthesia. One surgeon who preferred minimally invasive surgery did acknowledge that there may be advantages to open inguinal hernia repair under local anesthesia for some select groups of patients. There was also a concern that use of local anesthesia for open inguinal hernia repair had the potential to increase operative time relative to general anesthesia, which could undermine the advantages of local anesthesia for cognitive health.

One leader noted that even if surgeons might be willing to use local anesthesia for hernia repairs, some anesthesiologists may feel more comfortable with general anesthesia, possibly because this involves less workload. Another leader acknowledged one benefit to use of local anesthesia is that it may decrease the requirement for recovery in the post-anesthesia care unit, thus increasing the availability of this resource for patients undergoing more complex procedures.

## 4 | Discussion

Our study of older Veterans undergoing inguinal hernia repair and other stakeholders involved in this procedure identified several key barriers and facilitators to greater use of local anesthesia. The findings can serve as a roadmap for those seeking to follow guidelines that promote use of local anesthesia for older adults, who are more likely than younger adults to experience cognitive and physical decline following exposure to general anesthesia [7].

We found that the way surgical care was organized at the sites in our study did not allow for patients, surgeons, and anesthesiologists to have a joint conversation with shared decision-making about the advantages and disadvantages of local versus general anesthesia. This was reflected in several patients’ perceptions that the decisions about anesthesia type were made without patient involvement. This was also reflected in anesthesiologists’ perceptions that surgeons and patients often made decisions about anesthesia type—or set patient expectations one way or the other in a way that was difficult to reverse—without allowing the anesthesiologist to contribute to the conversation. A frequent theme that emerged in our discussions with stakeholders was how choice of anesthesia modality is difficult to disentangle from the selection of surgical approach, since minimally invasive surgery requires use of general anesthesia, and many surgeons feel very strongly that minimally invasive hernia repair is superior to the open technique. Consequently, as the use of minimally invasive approaches for hernia surgery has proliferated, fewer surgeons are comfortable with open techniques, much less open repair while using local anesthesia. Although some surgeons may perceive that minimally invasive repair is superior to open surgery from the standpoint of chronic pain or hernia recurrence, these benefits should be weighed against the potential cognitive and recovery benefits of local anesthesia, especially for older adults [19].

Our findings highlighted how surgeons are at the epicenter of the choice between local and general anesthesia—not just the selection of surgical technique. Anesthesiologists typically meet patients after the surgeon has made a decision to use a minimally invasive approach, which dictates the use of general anesthesia. In addition, even if the surgeon has chosen open surgery, they may have inadvertently precluded further conversation about different anesthesia techniques by telling patients that they will be asleep for the operation.

Beyond the surgeon’s role in selection of anesthesia type, we found that institutional factors also played a large role in the underutilization of local anesthesia. Standard pre-operative care workflows often did not allow for shared decision-making that incorporated all perspectives, and there was a lack of standardized protocols that would make local anesthesia easier to implement. A lack of directives from senior leadership to consider alternative anesthesia approaches also contributed.

A pervasive theme among the surgeons and other stakeholders that we interviewed was the feeling that the evidence of benefit for local over general anesthesia for older patients was not entirely clear, and that the benefits of minimally invasive surgery outweighed the advantages of local anesthesia. Despite ample evidence that using local anesthesia as an alternative to general anesthesia for inguinal hernia repair leads to outcomes and costs at least as good or better than using general anesthesia [8–11], relative equivalence is unlikely to be compelling enough to convince surgeons to more frequently discuss and/or use local anesthesia for their patients. Importantly, studies comparing local versus general anesthesia included mostly younger and healthier patients, and none included a comparison group having minimally invasive surgery. Although our research group published a large observational study showing that open surgery with local anesthesia offers similar morbidity but greatly reduced operative and recovery time compared to minimally invasive surgery [6], more convincing evidence in the form of a randomized trial focused on older adults and including a minimally invasive arm would be far more persuasive.

### 4.1 | Roadmap for Guideline-Aligned Use of Local Anesthesia for Hernia Repair in Older Adults

Efforts to increase the use of local anesthesia for hernia repair in older adults will require a multi-component approach. We posit that the first step should be conversations with surgeons about the potential advantages of local anesthesia for older adults. Importantly, these advantages should not be presented as directly conflicting with the advantages of minimally invasive surgery. Surgeons should be able to recognize the important advantages and disadvantages regarding both surgical approach and anesthesia type, and these decisions should be personalized for every patient. A second step toward increasing the use of local anesthesia could be the creation of institutional structures that support the use of local anesthesia. These structures could include opportunities for conversations with anesthesiologists earlier in the pre-operative care workflow and the creation of standardized protocols for the logistical aspects of the use of local anesthesia, such as clarifying who is responsible for performing blocks and specific instructions for the dosing and administration of anesthetic medications. Hospital leaders have the resources and power to implement policies, protocols, training, and infrastructure necessary to change practice, and our findings demonstrated that leadership buy-in is crucial for any changes to occur. Finally, the availability of high-quality, level-one evidence about the benefits of local anesthesia for inguinal hernia repair in older adults could serve as a strong impetus for using this approach. Since the published studies have not adequately explored outcomes for older patients, it would be reasonable to consider a new randomized trial focused specifically on older adults.

Our study had several limitations. First, since this was a convenience sample of VA hospitals that performed inguinal hernia repair under both local and general anesthesia, the results from these hospitals may not generalize outside this setting. Although the rate of local anesthesia in this study (47.5%) exceeded the national rate (10%–15% [12, 13]), which may indicate selection bias, we sought out these specific VA hospitals so we could achieve the requisite sample size of patients who underwent local anesthesia. In addition, our prior work found that the majority of inguinal hernia operations performed at the VA around the time of the study were performed using an open technique [20]. Additionally, we selected hospitals that perform some hernia operations with local anesthesia, so there may be additional barriers for facilities that currently perform little or no surgery using local anesthesia. Second, older Veterans likely differ in some ways from older non-Veterans, who may elucidate new or complementary barriers and facilitators.

## 5 | Conclusion

Our study provides insight into why the use of local anesthesia for hernia repair has declined over the preceding decades and highlights potential opportunities to reverse this trend. A multi-component implementation plan could increase the use of local anesthesia with clear advantages for older patients and health systems.

## Supplementary Material

1

2

3

Additional supporting information can be found online in the Supporting Information section. File S1: Patient interview guide. File S2: Interview codebook (patients). File S3: Interview codebook (providers).

## Figures and Tables

**TABLE 1 |:** Themes and example quotes from patients and providers regarding use of local anesthesia for inguinal hernia repair.

COM-B framework component	Study participant group	Theme identified by study participants	Example veteran quote(s)
Capability	Patients	Lack of understanding about the risks and benefits of local versus general anesthesia	“I have no medical knowledge, so I wouldn’t know one anesthesia from another, but … they said they were going to do such-and-such with the anesthesia, and I said, okay, well, you just go right ahead because I don’t know one from the other.”
		Hesitancy to ask questions and placements of blind trust in providers, limiting shared decision-making	“… it would probably be, you know, would you prefer Supercalifragilisticexpialidocious or (gibberish) (laughs)? You know, and I don’t even know what they’re talking about … I mean, they were in charge. I wasn’t trying to ask a bunch of stupid questions or tell them how to do their job.”
	Providers	Lack of surgeon comfort with local anesthesia	“Probably surgeon reluctance and surgeon practice [are barriers to local] … if you have surgeons that are used to … general anesthesia for open inguinal hernia repairs, … convincing them to change that practice and move toward local knowing that, … they may encounter more patient movement and it may be more technically challenging to do patients under local as compared to general.”
Opportunity	Patients	No opportunity for shared decision-making in standard pre-operative care workflows	“… the dude that came in and told me, he’s going to use a local. That was the first time I was a little bit concerned because I wanted to be out. But he told me he going to use local and I’d feel better, and I’d thank him for it when I get out of surgery because I wouldn’t have a headache later on in the day.” “He said, well, I don’t like your EKG. I don’t like the way your heart looks. You’ve got a valve there. And he said, I’m just going to do a local.”
	Providers	Lack of standardized protocols for use of local anesthesia	“Well, just logistically, they would have to define who does the regional block. Is it the surgeon, because in some centers, the surgeon does them, or is it the anesthesiologist? And when and where is it done?”
		Limited opportunity for anesthesiologists to participate in shared decision-making	“A lot of it helps if it comes from the surgeon themselves when … they provide expectations for the patient and the procedure itself and what the OR experience is. I think that helps a lot, rather than us coming in there and saying, hey, we can do this under local … If the surgeon is behind it … I do believe that helps quite a bit.”
		Lack of buy-in from senior leadership	“It would have to come, you know, from, and the chief of staff and then the head of surgery … really standing behind this.”
Motivation	Patients	Prior experience undergoing surgery drives openness to use of local anesthesia	“… anesthesia type wasn’t really something I concentrated on. But, yeah, it was mentioned. But, you know, … I’ve had a lot of surgeries, so …”
		Concern about side effects from general anesthesia leads to openness to use of local anesthesia	“Actually, I had more trouble with not just urination … I was constipated. And I took, I didn’t go to the bathroom for 3 days, and I was taking a laxative. Now every time I’ve ever had anesthesia, it makes it very difficult for me to urinate, very. It’s so bad that one time I did an outpatient … I had to go back to the hospital and have them catheter it.”
		Providing time for patients to voice their concerns may increase patients’ confidence in choosing local anesthesia	“… we have to try to make them comfortable, so … we always let them know that … if you’re going to be awake, you’ll certainly be in a capacity to let me know if you’re uncomfortable in any way. And I’m going to be there to make sure that we stop, get you comfortable, and move forward. You know, so there’s some reassurances that have to take place, but if the surgeon sets the tone from the very beginning, it’s very, very helpful.”
		Providers perceived that patients’ prior experiences with surgery could be major barriers to use of local anesthesia	“Number one barrier is patient. Like if patient doesn’t want that, patient wants to go to sleep, that’s the number one barrier …”
	Providers	Surgeons’ preference for robotic and laparoscopic approach necessitates general anesthesia, but some recognition of the benefits of local anesthesia for certain patients	“I think that, you know, that there are a lot of people who like doing minimally invasive surgery for this. They’re more comfortable with it. And whether it’s right or wrong, whether the data is there for it or not … they think that the outcomes are probably better.” “… my preferred approach is MIS, which will be under general, but there [are] some clear and convincing arguments for local having its advantages.”
		Concern that use of local anesthesia increases operative time	“… whereas you could have done the same operation, maybe under general still but open, in a much shorter period of time.”
		Anesthesiologists may prefer general anesthesia because this results in reduced workload	“some anesthesiologists that might rather the patient be asleep than awake in the case (laughs).”
		Local anesthesia may address capacity issues post-anesthesia care units	“… A nurse might actually rather it be local, again, because of the post op needs, it might be easier on nursing. Facility wide … there was the issue in there about PACU or non-PACU for post op. That is probably a benefit there, especially if your facility doing a lot of other complex cases at the same time, you don’t want to fill up your, your PACU with some of the more basic, easier cases if you can help it …”

**TABLE 2 |:** Veteran characteristics.

	Veterans
Total *n*	40
Age (mean, SD)	72.9 (5.2)
Race	
Black/African American	13 (32.5%)
White	26 (65%)
Unanswered	1 (2.5%)
Ethnicity	
Non-Hispanic	40 (100%)
Surgical approach	
Open	38 (95%)
Robotic	2 (5%)
Anesthesia modality	
General	18 (45%)
Local	19 (47.5%)
Converted	3 (7.5%)

**TABLE 3 |:** Provider (surgeon, anesthesiologist, and leadership) characteristics.

	Surgeons	Anesthesiologists	Hospital leaders
Total N	10	9	7
Age			
< 45 years	6 (60)	3 (33)	5 (71)
45–60 years	3 (30)	5 (55)	1 (14)
> 60 years	1 (10)	1 (11)	1 (14)
Race			
Asian	2 (20)	7 (78)	0 (0)
White	8 (80)	2 (22)	7 (100)
Black	0 (0)	0 (0)	0 (0)
Ethnicity			
Hispanic	0 (0)	0 (0)	0 (0)
Non-Hispanic	10 (100)	9 (100)	7 (100)
Gender			
Female	1 (10)	6 (67)	1 (14)
Male	9 (90)	3 (33)	6 (86)
Fellowship trained	10 (100)	6 (67)	5 (71)
Years in practice			
< 5 years	3 (30)	1 (11)	0 (0)
5–10 years	2 (20)	1 (11)	0 (0)
10–19 years	3 (30)	2 (22)	0 (0)
> 20 years	2 (20)	5 (56)	6 (86)
Not applicable	—	—	1 (14)

*Note:* All numbers represent *n* (%).

## Summary

- Key Points
  - Even though use of local anesthesia for inguinal hernia repair in older adults has clear advantages for cognitive function and post-operative recovery, local anesthesia is used far less frequently in real-world practice than general anesthesia.
  - We identified multiple barriers and facilitators to increasing the use of local anesthesia for inguinal hernia repair in older adults.
  - We found that patients were often not consistently offered the opportunity to have a shared decision-making conversation prior to surgery that incorporated the perspectives of all providers, including anesthesiologists, because of entrenched beliefs about the benefits of minimally invasive surgery and institutional structures that supported the use of general anesthesia.
- Why Does This Paper Matter?
  - Our results can be used to support a multi-component intervention plan to increase the use of local anesthesia for inguinal hernia repair in older adults, with key advantages for post-operative cognitive and functional recovery.
