# Supplementary material for: Barriers to the Implementation of Local Anesthesia for Older Adults Undergoing Inguinal Hernia Repair
Source: J Am Geriatr Soc. Author manuscript; Available in PMC 2026 Sep 11. (PMC13563494; doi:10.1111/jgs.70402)
Supplement: Supplementary file 1 [file NIHMS2206395-supplement-3.pdf]

Supplemental Material: Patient Interview Guide

Barriers to the Implementation of Local Anesthesia for Older Adults undergoing Inguinal Hernia Repair

Mai and Cher et al., 2026

Date:
Participant #:
Interviewer:
Anesthesia Type:

Patient Interview Guide

Interview Consent

Introduction:

Thank you for taking time to speak with us today. My name is ___________ and I am a researcher conducting interviews to learn more about your experience with hernia surgery.

- We are interested in learning more about the best way to fix groin (inguinal) hernias and ensure that patients like you have the best recovery possible from the procedure. You had a groin hernia fixed at our VA and we would like to learn more about your experience with the surgery and recovering from surgery.
- This interview will take about an hour and will have three parts: 1) asking you about your experience of hernia surgery; 2) asking you your opinion about a range of side effects that can happen with hernia surgery; and 3) asking you your thoughts about clinical trials.
- This interview is about your thoughts and experiences, so there are no wrong answers – we are interested in learning about how you felt after the surgery and what we can do to make that experience better.
- Is that ok with YOU

Confidentiality:

- Anything you say to me is completely confidential and your name and any identifying information will not be used in any reports or publications.
- Although I will ask to record this interview, the audio will be transcribed and de-identified by a professional transcription firm. The audio will then be destroyed.

- Is it ok if I continue to record? **[note to interviewer: if respondent agrees, keep recorder on. If respondent declines, take detailed notes of interview and stop recording]**

Rights:

- If any questions make you uncomfortable, you can choose not to answer them.
- You can choose also to end the interview at any time.
- You will receive a $50 check from the VA as a "thank you" for your participation.

Is that okay with you?

Great! Let's start.

QUESTIONS	AIMS AND INTERVIEWER NOTES
**A. <u>Overall Experience with Hernias and Surgeries</u>**<br><br>1) I would like to talk about your experience when you realized that you had a hernia.<br>a) When did you first realize you had a hernia?<br>i) ***[If patient noticed:]*** How did it feel?<br>ii) ***[If Dr. noticed:]*** What they tell you about it?<br><br>2) Did you have any concerns about having it repaired?<br>a) ***[If yes]*** What were you concerned about?<br><br>3) Tell me about your first meeting with your surgeon.<br>a) What do you remember about what they told you to expect for this surgery?<br>b) What did they say about how to prepare?<br>c) Did they tell you anything specific about the type of anesthesia you might be getting?<br>i) ***[if yes:]*** What were your thoughts about what kind of anesthesia you would prefer?<br>d) What did they tell you about what to expect during recovery?<br>e) Did you have any questions for them at that time?	***Aim: Understand the diagnosis process and how the patient prepared for surgery***
**B. <u>Previous Experience with Hernias and Surgeries</u>**<br><br>4) Before we go to much further into discussing this surgery, I'd like to know about any previous experiences you've had with surgery.	***Aim: Briefly understand participant's overall experience with hernias and other surgeries.***

a) What kinds of surgeries have you had prior to this one? ***[If any prior surgeries]***<br>i) Prior to this surgery, what was your most challenging surgery?<br>(1) What about it was challenging?<br>ii) Prior to this surgery, what would you say was your easiest previous surgery?<br>(1) What made it an easy surgery?<br><br>b) This study focuses on the use of anesthesia in surgery. What was your experience with anesthesia during those surgeries?<br>***Prompts:***<br>i) What type did you have?<br>ii) Did you have any issues or concerns?<br>c) Thinking back on those surgeries, did you have any issues with:<br>***Prompts:***<br>(1) Pain<br>(2) Fogginess or confusion<br>(3) Difficulty getting mobile again<br><br>d) How did those experiences with surgery affect how you thought about this surgery?<br><br>5) ***[if not mentioned:] Have*** you had any prior <u>hernia</u> surgeries? ***[If yes]***<br>a) How did those go?<br>b) What about the earlier hernia surgeries was challenging?<br><br>6) Based on what you've told me about your experience with surgeries prior to this, what concerns did you have going into this surgery?<br><br>7) In thinking ahead to this hernia surgery, did you talk about the surgery with anyone such as family or friends?<br>a) ***[if yes:]*** What advice did they offer?	
**C. <u>Day of Surgery Experiences</u>**<br><br>9) Now let's go back to the <u>day of this</u> surgery, can you walk me through that day?<br>a. Did you have any concerns going into the surgery?<br>i. ***[if yes:]*** What were they?	***Aim: understand the experience of the day of surgery, any concerns, any negative or positive experiences on that day.***

b. ***[if concerns].*** Were you able to ask doctors or nurses or anesthesiologists about your concerns?<br>i. ***[if yes:]*** What did they tell you?<br>ii. How did you feel after their response?<br><br>c. Normally, there are one of two types of anesthesia given for a hernia surgery. The first type, local anesthesia, numbs the part of the body where the surgeon is operating. The surgeon will also give you medicine that will make you fall asleep, so you don't feel anything during the operation. The second type, general anesthesia, puts you all the way to sleep, and also temporarily paralyzes you, so the doctor needs to put in a tube to help you breathe and that comes out at the end of the operation. Were you told about the type of anesthesia you would be receiving?<br>i. ***[if yes]*** What kind did you have?<br>ii. ***[if incorrect]*** Would you be surprised to know that our records show that you had **[Local/General]**?<br>**1.** How does that make you feel?<br>**2.** What made you think it was not **[Local/General]?**<br><br>10) Can you tell me what you remember about the surgery itself?<br><br>10 cont.…<br>***[especially with pts who received local]***<br>a. Do you remember any pain or discomfort during the operation?<br>b. Did you about any concerns about waking up or being awake during the surgery?<br><br>11) Can you take me through the rest of the day after your surgery was done?<br>a. What do you remember happening in the hospital after your surgery?<br>b. What do you remember about the discharge process?<br><br>12) What do you remember about the ride home? Any discomfort or issues in that process?	

D. <u>Post-surgical Recovery at Home:</u>

Aim: Questions aimed at understanding how the patient felt during their recovery at home.

13) What was the first night at home like?
   a) Any pain?
   b) Difficulty sleeping?
   c) Feeling foggy or confused?
   d) Difficulty moving around?
   e) Any difficulty with urination?
   f) Taking care of yourself?

14) What about your first day at home - were there any problems during that first day after surgery?
   a) ***[if yes:]*** What were they?
      i) Pain?
      ii) Feeling foggy or confused?
      iii) Difficulty moving around?
      iv) Any difficulty with urination?
      v) Taking care of yourself?

   b) What did you do to cope with those problems?
      [Prompts:]
      (1) Call clinic
      (2) Ask friends/family for help
      (3) Seek online information

15) Let's talk about the first few weeks at home after your surgery: when recovering from surgery, sometimes things come up that we didn't expect.
   a) Were there any surprises or things you didn't expect that happened after the surgery?
   b) Were there any challenges or difficulties that you experienced?
   c) Were there any pleasant surprises?

16) How back to your "normal" activity levels are you since having your surgery?
   a) What does back to "normal" mean for you?
      i) Does that include what you couldn't do when the hernia was there?
      ii) What about prior to having the hernia repaired?
   b) When, in the process of your post-surgical recovery, would you say you got "back to normal?"

Aim: to understand 1) how back to baseline are they? 2) what was baseline (pre-hernia or with the hernia and pre-surgery)? 3) at what point following the surgery did they feel they were at baseline or close to baseline?

**<u>F. Responding to Potential Surgical Outcomes</u>**<br><br>Thank you for telling me about your experience with surgery. Now we are going to talk about some "what if?" situations and ask you to give us your opinion on those.<br><br>**Some patients may experience complications during or after surgery. This can happen because of the surgery itself. For example, the surgeon can cause an injury while doing the operation. Complications can also happen because you are getting anesthesia and other medications during the surgery that can have side effects, or because you had a breathing tube placed.**<br><br>I'm going to describe some of the experiences that <u>some</u> patients may have during or after hernia repair surgery. We'd like to know how you might feel, these experiences happened to you. I'm going to tell you about each potential experience and then ask you to rate how much each experience would <u>bother</u> you if it happened to you, on a scale of 1-10.<br><br>If something wouldn't bother you at all or would only bother you a little, I'll ask you to give it a 1. If it would bother you a lot, say, you might be very concerned if that happened to you, I'll ask you to give it a 10. And if it would bother you some, but not a lot, pick a number in between 1 and 10. Ok?<br><br>17) **Sometimes, when the hernia repair surgery is done using numbing medication as opposed to putting people all the way to sleep with a breathing tube, they experience some pain or discomfort *during* the surgery and the surgeon or anesthesiologist will give them additional medication.**<br>a) If this were to happen to you, how would you feel?<br>b) Would that bother you just a little or not at all – for a score of 1, or bother you a lot for a score of 10, or in between?<br>c) Can you tell me more about why you gave it the score you did?<br><br>18) **After having a hernia repair surgery, some patients who had a breathing tube while they were asleep may have a sore throat that can last a few days or longer.**<br>a) If this were to happen to you, how would you feel?	***Aim: Rate the implications from patient's perspective of some likely surgical outcomes on a scale of 1-10, ask why they rated as they did.***<br><br>***NOTE: text in bold must be read verbatim to the respondent.***<br><br>***Interviewer: if the patient asks, you may say that "the placement of the breathing tube can cause a sore throat through rubbing the inside of the throat during surgery."***

b) Would that bother you just a little or not at all – for a score of 1, or bother you a lot for a score of 10, or in between?<br>c) Can you tell me more about why you gave it the score you did?<br><br>19) **After having a hernia repair surgery, some patients who had a breathing tube may have an increase in congestion or feeling like they have stuff in their chest**<br>a) If this were to happen to you, how would you feel?<br>b) Would that bother you just a little or not at all – for a score of 1, or bother you a lot for a score of 10, or in between?<br>c) Can you tell me more about why you gave it the score you did?<br><br>20) **One of the things that can happen *after* a hernia repair surgery is that a person may have difficulty peeing or be unable to pee. If that happens, a surgeon may insert a catheter through the penis to help get the pee out. This may only need to be done once, or it may have to be left in the patient's bladder for several weeks.**<br>a) If this were to happen to you, how would you feel?<br>b) Would that bother you just a little or not at all – for a score of 1, or bother you a lot for a score of 10, or in between?<br>c) Can you tell me more about why you gave it the score you did?<br><br>21) **After having a hernia repair surgery, some patients notice changes in their ability to think or remember, trouble understanding, or trouble reasoning through things. This may be something that the patient notices, or it may be something that they don't notice, but their family or friends notice.**<br>a) If this were to happen to you, how would you feel?<br>b) Would that bother you just a little or not at all – for a score of 1, or bother you a lot for a score of 10, or in between?<br>c) Can you tell me more about why you gave it the score you did?<br><br>22) **After having a hernia repair surgery, some patients have pain after their surgery that lasts longer than they expected.**	***Interviewer: If asked, you can say that "sometimes fluid can collect in the lungs due to the breathing tube sometimes pushing stuff from the throat and mouth down into the lungs."***<br><br>***Interviewer: If asked why, it can be because anesthesia slows down the bladder.***<br><br>***Interviewer: If asked why, anesthesia can linger in the body longer than expected causing people to be a bit confused. Also, surgery stresses the body and brain and can cause some changes in ability to think and remember***

a) If this were to happen to you, how would you feel?
b) Would that bother you just a little or not at all – for a score of 1, or bother you a lot for a score of 10, or in between?
c) Can you tell me more about why you gave it the score you did?

23) **After having a hernia repair surgery, some patients may get an infection at the wound site.**
a) If this were to happen to you, how would you feel?
b) Would that be a one, not very concerning, or a ten, very concerning, or in between?
c) Why did you rank it that way?

24) **After having a hernia repair surgery, some patients find that they have difficulty getting back to normal levels of activity.**
a) **If it took one month for you to get back to your normal activity, how would you feel?**
b) Would that bother you just a little or not at all – for a score of 1, or bother you a lot for a score of 10, or in between?
c) Can you tell me more about why you gave it the score you did?

25) **If it took more than two months for you to get back to your normal activity, how would you feel?**
a) Would that bother you just a little or not at all – for a score of 1, or bother you a lot for a score of 10, or in between?
b) Can you tell me more about why you gave it the score you did?

26) **If it took more than three months for you to get back to your normal activity, how would you feel?**
a) Would that bother you just a little or not at all – for a score of 1, or bother you a lot for a score of 10, or in between?
b) Can you tell me more about why you gave it the score you did?

27) **The things we've just discussed are a list of the kinds of things that can go wrong during or after hernia surgery. Are there any other issues or concerns that you have experienced or heard about that you'd like to add to that list?**

If any mentioned:<br>a) What rating would you give that problem?<br>b) Why did you give it that score?<br><br>28) **Which of the experiences that we just discussed would bother you the most?**<br>a) Why?	
**E. <u>Clinical Trial Enrollment</u>**<br><br>Thank you for that important input. Now I'd like to talk with you about something a bit different. As you know, we want to figure out the best way to fix hernias in patients like you.<br><br>Normally, there are two types of studies that we recruit patients for. The first type is a research study, like the interview you are doing today, helps us learn more about patients' general experiences of anesthesia and hernia surgery. The second type is a clinical trial, where patients are randomly assigned a particular type of anesthesia before surgery and then their results are compared to see which approach is the best one.<br><br>**Any questions about the differences between those two types of research?**<br><br>We are thinking about doing a clinical trial in the future comparing patients who receive both local anesthesia and general anesthesia. We are not trying to recruit you for the clinical trial but are trying to learn about what makes patients interested, or not interested, in participating in a clinical trial.<br><br>29) Thinking ahead to the future, if you were asked to participate in this kind of clinical trial, would you participate?<br>***Prompts:***<br>a. What would make you most interested in participating in a clinical trial?<br>b. what might make you hesitate to participate in a clinical trial?<br>**i. [If participant mentions conspiracy theories]** Interesting – where did you hear that?	***Aim: Gleaning information from patients about ways to increase enrollment and improve experience for the trial.***<br><br>***Note: If participant becomes agitated or angry in response to questions 29-31: SKIP 32 and 33***

30) Have you ever participated in a clinical trial in the past?<br>***[If yes]***<br>a) What was that like for you?<br><br>31) Have any friends or family members participated in a trial?<br>***[If yes:]***<br>a) What did they tell you about it?<br><br>32) I'm going to list some of the reasons people enroll in clinical trials. Can you let me know if they would make you more likely to enroll in the future?<br>***Prompts:***<br>a) Some people want to enroll in clinical trials because they feel like it will give them access to the newest treatments.<br>b) Some people enroll in clinical trials because they feel like it will give them more face to face time with the doctors who are running the trial.<br>c) Some people enroll in clinical trials because usually trial participants are compensated financially.<br>d) Some people enroll in clinical trials because they want to help other people who might have the same condition at a later point in time.<br><br>33) I'm going to suggest some reasons that some people don't want to enroll, and you can let me know if you would agree. Some people say they would rather not be in a clinical trial because they:<br>***Prompts:***<br>a) Are concerned that they might be assigned a lesser treatment<br>b) Don't want to go to the hospital more frequently or would prefer to have virtual visits rather than in-person visits<br>c) Think it seems like it might take a lot of time<br>d) Don't like experiments in general	
**F. <u>Wrap up</u>**<br><br>34) Now that you've had groin hernia surgery yourself, what advice would you give to another patient having the same operation?	***Aim: Elicit any additional information not provided in interview.***

35) Do you have any other comments or thoughts on this topic that I haven't asked about?<br><br>36) Is there anything I haven't asked you that you think I should have?<br><br><br><br><br>Great! Our interview is over, and I appreciate your time very much. I'll let the team at the VA know that we have completed the interview and they will send you a $50 check in the mail from the VA within the next couple of weeks in appreciation for your time.	

Time Completed:___________

Initials:____________________

Outcome Ranking Sheet

Inguinal Hernia Surgery Possible Outcomes	Importance
Pain or discomfort *during* the surgery	
Sore throat lasting a few days or longer *after* the surgery	
Increase in chest congestion or trouble breathing *after* the surgery	
Difficulty peeing or unable to pee *after* the surgery	
Changes in ability to think or remember *after* the surgery	
Pain lasting longer than expected *after* the surgery	
Difficulty getting back to normal activity levels *after* the surgery (see below):	
1 month	
2 months	
3 months	
Infections at the wound site *after* the surgery	
**Others (add below):**	

Instructions: Ask the participant to rate how bothersome the outcome would be to them on a scale of one to ten, listing the number in the right-hand column. Also add any other outcomes that participants think are important in the rows below "others":

1. Would bother participant a **little** or not at all
2-9. Would bother participant some (varies)
10. Would bother participant **a LOT**
